# Protonation tuning of quantum interference in azulene-type single-molecule junctions†
†Electronic supplementary information (ESI) available: Synthesis and characterization of compounds, details of single-molecule conductance measurement and the calculation. See DOI: 10.1039/c7sc01014a



**DOI:** 10.1039/c7sc01014a

**Published:** 2017-09-07

**Authors:** Guogang Yang, Sara Sangtarash, Zitong Liu, Xiaohui Li, Hatef Sadeghi, Zhibing Tan, Ruihao Li, Jueting Zheng, Xiaobiao Dong, Junyang Liu, Yang Yang, Jia Shi, Zongyuan Xiao, Guanxin Zhang, Colin Lambert, Wenjing Hong, Deqing Zhang

**Affiliations:** a State Key Laboratory of Physical Chemistry of Solid Surfaces , iChEM , Department of Chemical and Biochemical Engineering , College of Chemistry and Chemical Engineering , Xiamen University , Xiamen 361005 , China . Email: whong@xmu.edu.cn; b Department of Physics , Lancaster University , Lancaster LA1 4YB , UK . Email: c.lambert@lancaster.ac.uk; c Organic Solids Laboratory , Institute of Chemistry , Chinese Academy of Sciences , Beijing 100190 , China . Email: zitong_@iccas.ac.cn ; Email: dqzhang@iccas.ac.cn

## Abstract

The protonation of azulene derivatives with quantum interference effects is studied by the conductance measurements of single-molecule junctions. Three azulene derivatives with different connectivities are synthesized and reacted with trifluoroacetic acid to form the protonated states. It is found that the protonated azulene molecular junctions produce more than one order of magnitude higher conductance than the neutral states, while the molecules with destructive interference show more significant changes. These experimental observations are supported by our recently-developed parameter free theory of connectivity, which suggests that the largest conductance change occurs when destructive interference near the Fermi energy in the neutral state is alleviated by protonation.

## Abstract 2

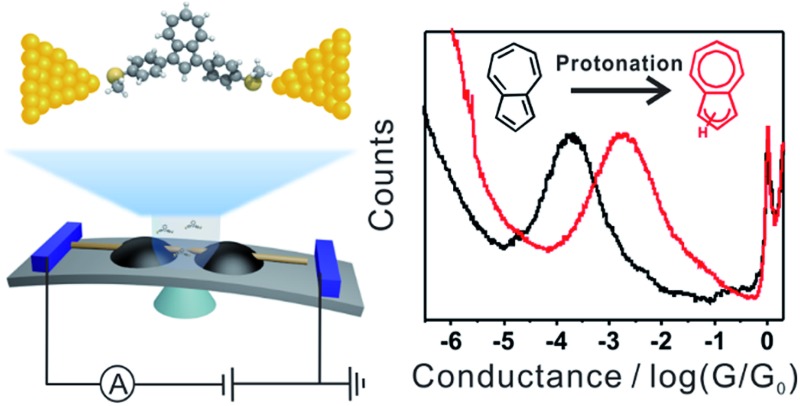
The protonation of azulene cores offers significant conductance tuning in single-molecule junctions with quantum interference.

## Introduction

The coupling of π-systems to each other is essential to their charge transport applications in organic and molecular electronics.^1–4^ Recently, quantum interference effects due to different electron pathways through molecular systems with varied connectivities have been intensively explored *via* single-molecule conductance studies of benzene,^5^ oligo(phenylene ethynylene),^6^ anthraquinone,^7^ unsaturated carbon chains,^8^ and azulene.^9,10^ Destructive quantum interference brings anti-resonances within their HOMO–LUMO gaps, which significantly reduces their single-molecule conductance and leads to new control strategies for molecular-scale devices.

Among those molecules exhibiting destructive quantum interference, azulene derivatives have also been considered as responsive molecular devices and materials,^11–13^ because the five-seven ring system of azulene undergoes protonation in response to acid.^14^ During protonation, the HOMO of the protonated azulene core with unpaired valence electrons is expected to move towards the Fermi level of the electrodes, which could create significant electrical conductance changes.^15,16^ Since the tuning of quantum interference is one of the major challenges for current molecular electronics,^10,17–20^ protonation studies of azulene derivatives may offer a unique opportunity for tuning charge transport through single-molecule junctions.

Here we studied the protonation tuning of charge transport through single-molecule junctions of several azulene derivatives using a mechanically controllable break junction (MCBJ) technique. The single-molecule conductance measurements demonstrate that the azulene derivatives showed significant conductance changes after adding trifluoroacetic acid (TFA). Furthermore, it is also found that the derivatives exhibiting destructive interference in the neutral state undergo more significant changes upon protonation. These measured trends can be understood using a parameter free theory to model the molecular junction and compute the electron transmission of azulene derivatives in their neutral and protonated states.

**Scheme 1:**
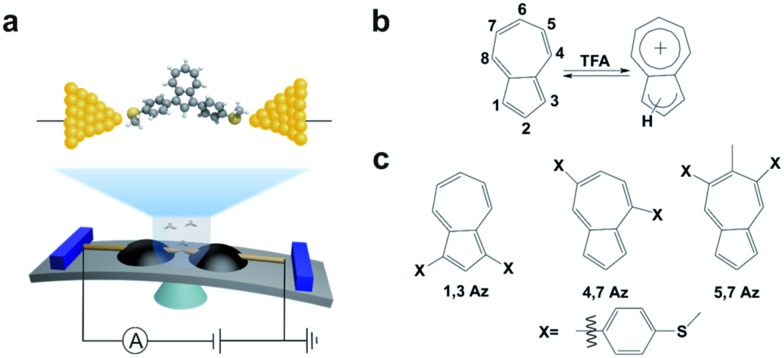
(a) Schematic of the MCBJ and the molecular junction of **1,3Az**. (b) The protonation process of azulene after adding TFA. (c) Azulene derivatives studied in this work.

## Results and discussion

**Fig. 1:**
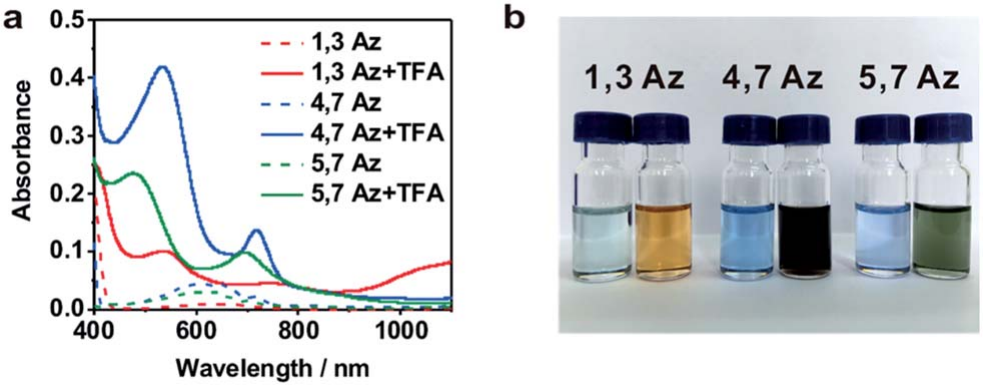
(a) UV-vis spectra of **1,3Az**, **4,7Az**, and **5,7Az** with/without TFA. (b) Color changes of **Az** solutions before (left) and after (right) adding TFA.

The azulene derivatives of **1,3Az**, **4,7Az** and **5,7Az** are designed with – SMe anchors with different connectivities to the azulene core, and their synthesis followed the synthetic route reported in ref. 21 and 22. UV-vis measurements were carried out for the azulene molecules dissolved in THF/TMB solution. After adding TFA, significant color changes from aquamarine blue to orange occurred (Fig. 1b), which is reflected in the appearance of the new peak at 540 nm and 745 nm, respectively, suggesting the presence of protonated **1,3Az** in solution. The new peaks also appear in the protonation of **4,7Az** (536 nm and 718 nm) and **5,7Az** (480 nm and 695 nm). The protonation of azulene could lead to the formation of stable tropylium cations, which causes significantly different properties compared with the neutral species.^23–27^

**Fig. 2:**
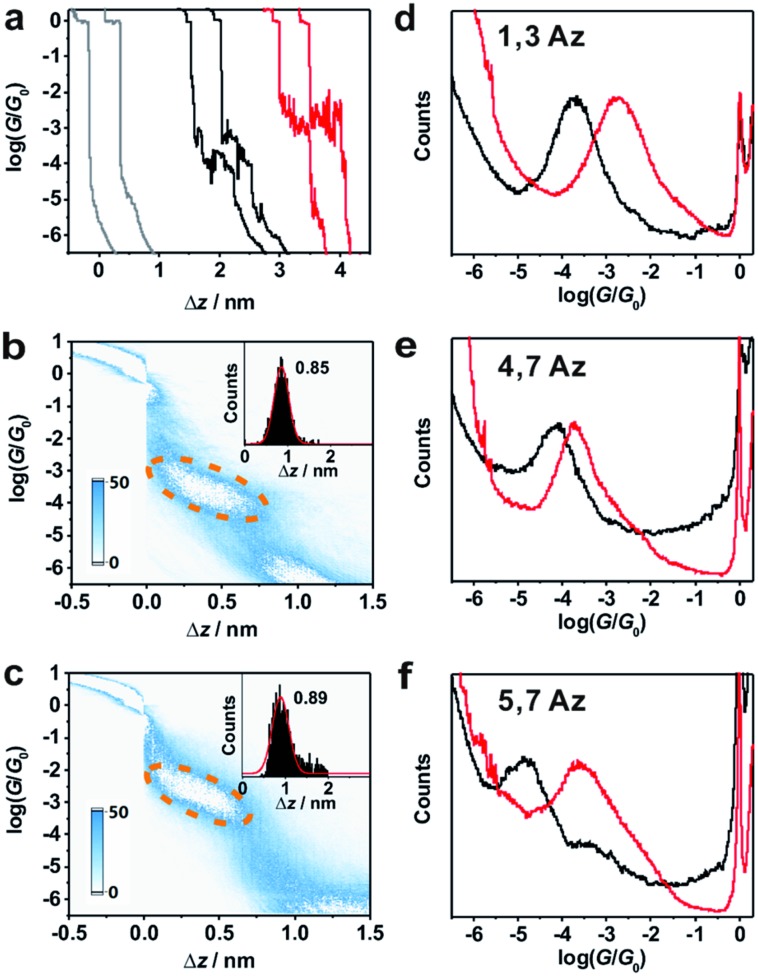
(a) Typical individual traces for single-molecule conductance measurement of **1,3Az** without (black) and with (red) TFA, and the blank (gray). 2D conductance histograms and stretched distance distributions (inset) for **1,3Az** (b) without and (c) with TFA. (d–f) Conductance histograms of **1,3Az**, **4,7Az** and **5,7Az** without (black) and with (red) TFA.

The single-molecule conductance measurements of azulene derivatives were carried out in a solution containing 0.1 mM target molecule in a mixture of THF (tetrahydrofuran) : TMB (mesitylene) = 1 : 4 (v/v) using the MCBJ technique (see ESI† for more details).^28–30^ To trigger the protonation, 10 μL (13.5 M) TFA was added into a 190 μL 0.1 mM solution of azulene derivatives in the liquid cell of the MCBJ set-up. Fig. 2a shows several typical individual stretching traces from the measurements in pure solvent, with **1,3Az**, and with **1,3Az** and TFA, respectively. The solvent experiment shows a tunneling decay after rupture of the gold–gold atomic contact (a plateau at conductance quantum *G*_0_), while the measurement of the targeted molecules reveals additional clear molecular plateaus varying from 10^–2^ to 10^–5^ *G*_0_, which are assigned to the conductance features of the Au-molecule–Au junctions.

Around 2000 individual traces are used to construct conductance histograms without data selection for further analysis. Fig. 2b and c represent the two-dimensional (2D) conductance histograms of the initial neutral state and protonated state of **1,3Az**. It is found that the conductance intensity cloud shifts to a higher conductance regime after protonation, while the relative stretched distance distribution (shown in the inset) showed quite similar lengths. These findings suggest that the conductance changes come from the enhanced charge transport through protonated azulene cores rather than from the configuration changes.

Fig. 2d shows the 1D conductance histograms of **1,3Az** before and after adding TFA. There is a pronounced peak located at 10^–3.8^ *G*_0_ for the neutral **1,3Az**. Control experiments of **1,3Az** with different concentrations suggested that the conductance feature comes from the single-molecule junctions and that the peak position is independent of the concentration in solution (see Fig. S2-5 in ESI†). Compared with the conductance of the initial neutral state, the conductance of **1,3Az** in the final state increased by more than one order of magnitude from 10^–3.8^ *G*_0_ to 10^–2.7^ *G*_0_. The conductance of protonated **1,3Az** is similar to that of conjugated tolane dithiol (10^–2.7^ *G*_0_)^29^ and even higher than that of biphenyl dithiol molecules (10^–3.8^ *G*_0_),^31^ even though **1,3Az** has a significantly longer length. These findings suggested that the protonated azulene core has great potential as a highly conductive building block for future molecular electronics studies.

We also studied the conductance changes before and after adding TFA. Plotted as black curves in Fig. 2d–f, the most probable conductances of **1,3Az**, **4,7Az**, and **5,7Az** are identified to be 10^–3.8^ *G*_0_, 10^–4.2^ *G*_0_, and 10^–4.9^ *G*_0_, respectively. The conductance of **5,7Az** falls below that of **4,7Az** although **4,7Az** has a longer length, suggesting the existence of destructive quantum interference in the charge transport through neutral **5,7Az**.^9^ After adding TFA for protonation, significant conductance enhancement is observed for both **4,7Az** and **5,7Az** (Fig. S2-2 and S2-3 in ESI†), while the changes of **5,7Az** are the most pronounced among the three derivatives. Interestingly, it is found that the conductance of **5,7Az** increased from 10^–4.9^ *G*_0_ to 10^–3.6^ *G*_0_ after protonation, which was even higher than the conductance of protonated **4,7Az**.

**Fig. 3:**
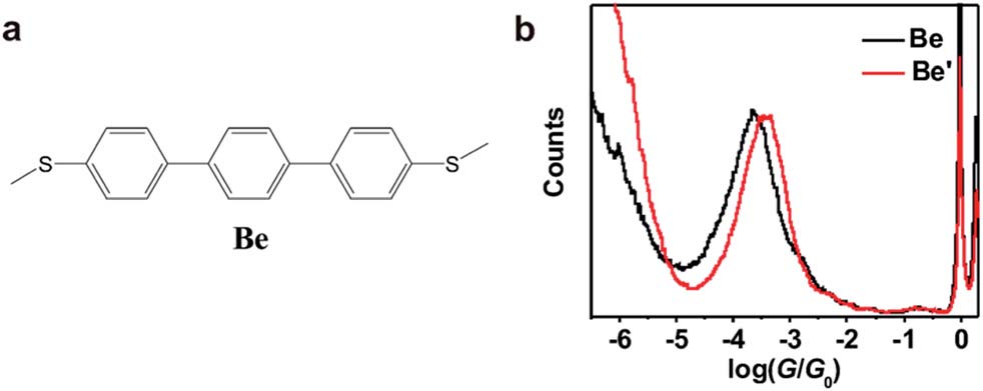
(a) Molecular structure of molecule **Be** for the control experiment. (b) Conductance histograms of molecule **Be** with (red) and without (black) TFA.

**Fig. 4:**
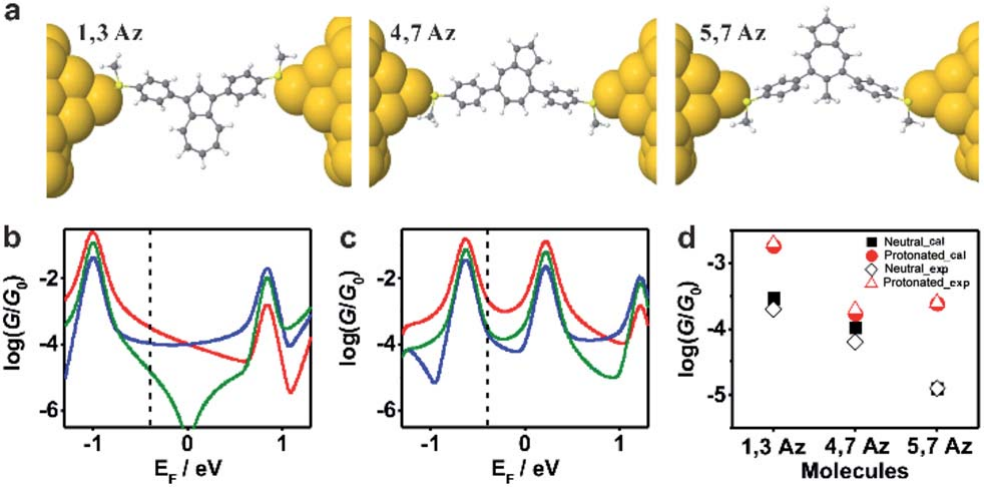
(a) Relaxed structures of the molecules from DFT. (b) Calculated conductance {*A*} of the initial state and (c) the protonated state for the molecules shown in Scheme 1c: **1,3Az** (red), **4,7Az** (blue), and **5,7Az** (green). (d) Comparison between the calculated conductance and the experimentally determined conductance. The transmission curves leading to these conductances are shown in Fig. S3-1 of the ESI.†

Table 1 summarizes the characterized molecular junction lengths from their plateau lengths (see ESI† for more details) and conductances with literature data for comparison. By adding the snap-back distance of 0.5 nm,^29^ the characterized lengths of the molecular junctions are 1.35 nm, 1.53 nm and 1.32 nm for **1,3Az**, **4,7Az** and **5,7Az**, and 1.39 nm, 1.51 nm, and 1.35 nm for protonated **1,3Az**, **4,7Az** and **5,7Az**, respectively. The agreement with the calculated molecular lengths (see also Fig. 4a) suggests that all of the molecules are able to be stretched to a fully-elongated configuration during the single-molecule conductance measurements. Furthermore, it is found that the conductances we determined for neutral azulene are qualitatively in good agreement with previous studies by Xia *et al.* ^21^ but quantitatively a difference of 10^–0.2^ *G*_0_ is obtained for each molecule. Control experiments in 1,2,4-trichlorobenzene (see Fig. S2-1 in ESI†) reveal that the quantitative difference comes from the solvent tuning effect in different solvents.^32,33^ To evaluate the environmental effect of adding TFA to the junctions, we carried out the control experiment with a similar molecule in which the azulene core is replaced by a phenyl core (Fig. 3a). A slight conductance increase from 10^–3.6^ *G*_0_ to 10^–3.4^ *G*_0_ is observed as shown in Fig. 3b (see Fig. S2-6† for 2D histograms), indicating that Fermi level shifting of the electrode^33–37^ by adding the TFA is not the major contribution of the conductance enhancement. A previous theoretical study suggests that the protonation could tune the destructive quantum interference in charge transport by changing the energy level of the molecular device.^20^ Thus, the significant conductance enhancement we observed for azulene junctions may come from the protonation of the azulene core.

**Table 1:** Molecular lengths and conductances determined from experimental measurements and DFT calculations

Molecule	Δ*z*_MCBJ_/nm	*z*_MCBJ_/nm	Calculated length/nm	log(*G*/*G*_0_) in the neutral state	log(*G*/*G*_0_) in the protonated state	log(*G*/*G*_0_) in ref. 21
**1,3Az**	0.85	1.35	1.39	–3.8	–2.7	–3.5
**4,7Az**	1.03	1.53	1.49	–4.2	–3.7	–4.1
**5,7Az**	0.82	1.32	1.28	–4.9	–3.6	–4.7

To further investigate the connectivity-dependence of conductance enhancement between the neutral state and final protonated state, we introduced a simple parameter free theory,^38–41^ in which the Hamiltonian H of the PAH core was identified with a connectivity matrix C, whose elements C_*ij*_ were equal to unity for *ij* nearest neighbors and zero otherwise. This theory correctly predicts conductance ratios for a range of PAHs and therefore here we use the same theory to investigate how the conductance of azulenes with different connectivities changes from the initial state to the final state. Starting from the azulene core Hamiltonian, the resulting room temperature electrical conductances for each connectivity^40^ are shown in Fig. 4b and c in the initial and protonated states. The neutral-state conductance (Fig. 4b) of the **5,7Az** connectivity shows destructive quantum interference near the gap center (*E*_F_ ≈ 0) over a wide energy range between –0.7 and 0.6. In contrast, the **4,7Az** and **1,3Az** connectivities show no such feature near the gap center and therefore their neutral-state conductance is higher than that of **5,7Az**. Comparing Fig. 4b with our measured conductances suggests that the Fermi energy in the neutral state is located just below the gap center (see the dotted black vertical line in Fig. 4b). With this choice of Fermi energy, the conductance order is **1,3Az** > **4,7Az** > **5,7Az** at the initial state (Fig. 4b).

In the protonated state, density functional theory reveals that the spin-up and spin-down energy levels of the azulene in a junction are not equal (Fig. S3-2 in the ESI†) and therefore the spin-independent transmission peaks associated with the neutral-molecule HOMO will split into separate spin-up and spin-down transmission resonances, leading to a reduction in the HOMO–LUMO gap of the protonated molecules. In addition, protonation is expected to introduce a negative electrostatic potential in the vicinity of the molecule, which causes the HOMO to increase in energy and move closer to the Fermi energy. (shown by the black vertical dashed lines in Fig. 4b and c). As shown in Fig. S3-2 in the ESI,† in the initial state the system is not spin polarised. However, as shown in Fig. S3-3–S3-5,† in the protonated state within the junction, due to the charge transfer from the molecule to the gold, spin-splitting occurs. The HOMO–LUMO gap in the protonated state arises from the spin-up HOMO and the spin down LUMO and consequently the new HOMO–LUMO gap in the protonated state is smaller than the initial one. In our tight-binding model, this splitting is taken into account using site energies which differ by 0.4 eV for spin up and down, respectively.

By choosing the Fermi energy in the tail of the LUMO, as shown in Fig. 4c, the conductance of all connectivities increases upon protonation, and the order is changed compared with that in the neutral state. In agreement with the experiments as shown in Fig. 4d, the conductance order is **1,3Az** > **4,7Az** > **5,7Az** in the neutral state and it changes to **1,3Az** > **5,7Az** > **4,7Az** in the protonated state. Uniquely, the neutral **5,7Az** possesses a sharp interference dip near the Fermi energy, and consequently exhibits the largest conductance increase upon protonation. In contrast, for **4,7Az** and **1,3Az**, the neutral state is not affected by destructive interference and therefore the shift towards the HOMO produces a smaller increase in conductance. Thus, the protonation of **5,7Az** provides more significant tuning than that of **4,7Az** and **1,3Az** (see ESI† for more detailed discussions).

## Conclusions

To conclude, we studied the protonation tuning of azulene derivatives with quantum interference effects *via* measuring the single-molecule conductance using the MCBJ technique. It is found that the single-molecule conductance of protonated azulene is more than one order of magnitude higher than the conductance of the initial state. More importantly, the molecule with destructive quantum interference near the Fermi energy at the neutral state could provide more significant conductance tuning. These experimental observations are supported by our recently developed parameter free theory. Our finding suggests that the protonated azulene derivatives could be considered as a promising building block for the construction of new conjugated materials with stimuli-responsive and enhanced charge transport ability, and reveals the potential of protonation as a novel approach to tune the quantum interference effect in charge transport at the single-molecule level.

## Conflicts of interest

There are no conflicts to declare.
