# Supplementary material for: Protonation tuning of quantum interference in azulene-type single-molecule junctions
Source: Chem Sci. 2017 Sep 7;8(11):7505–9. doi: 10.1039/c7sc01014a (PMC5676185; doi:10.1039/c7sc01014a)
Supplement: Supplementary file 1 [file SC-008-C7SC01014A-s001.pdf]

Electronic Supplementary Material (ESI) for Chemical Science.
This journal is © The Royal Society of Chemistry 2017

Supporting Information

Protonation Tuning of Quantum Interference in Azulene-Type Single-Molecule Junctions

Guogang Yang,‡[a] Sara Sangtarash,‡[b] Zitong Liu,[c,*] Xiaohui Li,[a] Hatef Sadeghi,[b] Zhibin Tan,[a] Ruihao Li,[a] Jueting Zheng,[a] Xiaobiao Dong,[c] Junyang Liu,[a] Yang Yang,[a] Jia Shi,[a] Zongyuan Xiao,[a] Guanxin Zhang,[c] Colin Lambert,[b,*] Wenjing Hong,[a,*] Deqing Zhang[c,*]

[a] State Key Laboratory of Physical Chemistry of Solid Surfaces, Collaborative Innovation Centre of Chemistry for Energy Materials, Department of Chemical and Biochemical Engineering, College of Chemistry and Chemical Engineering, Xiamen University, Xiamen 361005 (China)

[b] Department of physics, Lancaster University, Lancaster LA1 4YB (UK)

[c] Organic Solids Laboratory, Institute of Chemistry, Chinese Academy of Sciences, Beijing 100190 (China)

Abstract: The protonation of azulene derivatives with quantum interference effect is studied by the conductance measurements of single-molecule junctions. Three azulene derivatives with different connectivities are synthesized and reacted with trifluoroacetic acid to form the protonation state. It is found that the protonated azulene molecular junctions produce more than one order of magnitude higher conductance than the neutral states, while the molecules with destructive interference show more significant changes. These experimental observations are supported by our recently-developed parameter free theory of connectivity, which suggests that the largest conductance change occurs when destructive interference near the Fermi energy in the neutral state is alleviated by protonation.

1. Synthesis and characterization

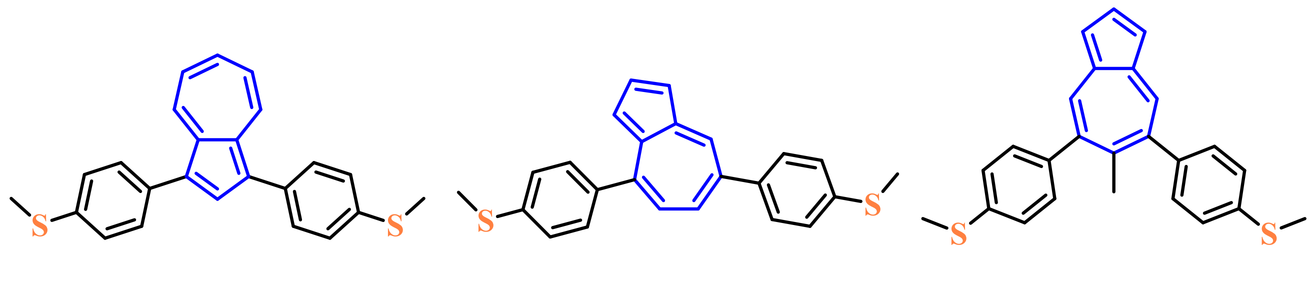

1,3 Az   4,7 Az   5,7 Az

Scheme S1 Chemical structures of azulene derivatives.

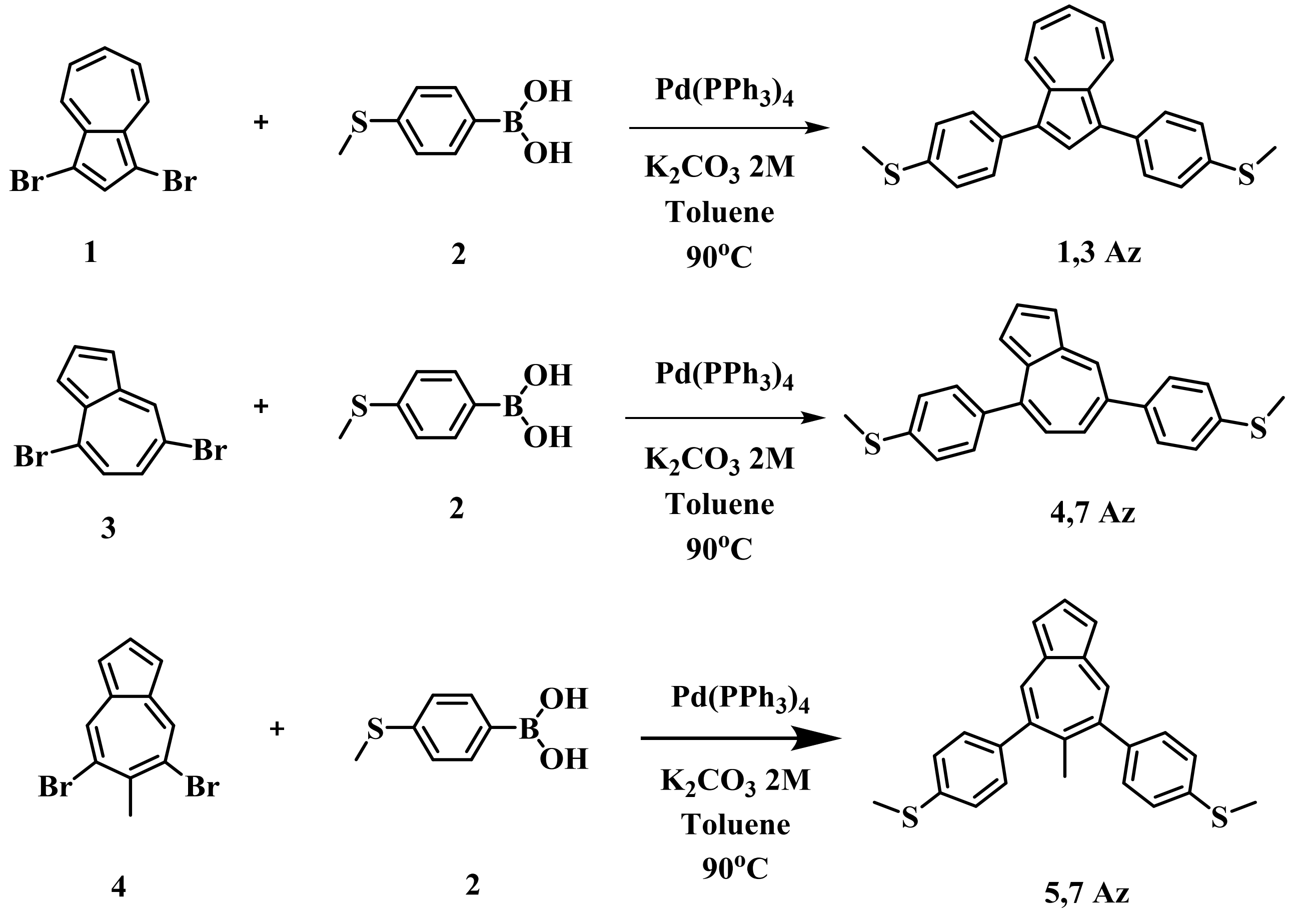

Scheme S2 Synthetic routes to azulene derivatives **1,3 Az**, **4,7 Az** and **5,7 Az**.

Materials and Characterization Techniques. The reagents and starting materials including compound **2** were commercially available and used without any further purification, if not specified elsewhere. Compounds **1**, **3** and **4** were synthesized according to the previous reports.[1, 2] ^{1}H NMR and ^{13}C NMR spectra were recorded on Bruker AVANCE III 400 and 300

MHz spectrometers. Elemental analysis was performed on a Carlo Erba model 1160 elemental analyzer.

Synthesis of 1,3-bis(4-(methylthio)phenyl)azulene (1,3 Az).

To a Schlenk flask equipped with a stir bar was added compound **1** (0.20 g, 0.70 mmol), compound **2** (0.25 g, 1.5 mmol), $Pd(PPh_3)_4$ (40 mg, 35 μmol). The vessel was sealed and evacuated/backfilled with nitrogen for three times, followed by the addition of dry toluene (10 mL) and a degassed 2.0 mL of aqueous solution of K_2CO_3 (2.0 M) via syringe. The mixture was heated at 90 °C for 24 h. After cooling, the organic layer was separated, concentrated, and redissolved in CH_2Cl_2 (20 mL). This CH_2Cl_2 solution was washed sequentially with water (10 mL) and brine (2 × 10 mL), and then dried over anhydrous Na_2SO_4. The crude product was purified by column chromatography on silica gel with petroleum ether (60-90 °C) and CH_2Cl_2 (8:1, *v*/*v*) as eluent. Compound **1,3 Az** was obtained as a green solid (164 mg) in 63% yield. ^{1}H NMR (CD_2Cl_2, 300 MHz): δ 8.50 (*d*, 2H, *J* = 9.6 Hz), 8.07 (*s*, 1H), 7.57-7.55 (m, 5H), 7.39 (*d*, 4H, *J* = 8.1 Hz), 7.14-7.12 (*m*, 2H), 2.55 (*s*, 6H). ^{13}C NMR (CD_2Cl_2; 75 MHz): δ 138.4, 136.0, 135.8, 135.3, 133.1, 129.3, 126.1, 122.9, 15.0. Anal. Calcd for $C_{24}H_{20}S_2$: C, 77.38; H, 5.41; S, 17.21. Found: C, 77.35; H, 5.42; S, 17.15.

Synthesis of (azulene-4,7-diylbis(4,1-phenylene))bis(methylsulfane) (4,7 Az).

4,7 Az was synthesized similarly as for **1,3 Az** with **3** (0.15 g, 0.52 mmol), compound **2** (0.19 g, 1.2 mmol) and $Pd(PPh_3)_4$ (30 mg, 26 μmol). Compound **4,7 Az** was obtained as a dark blue solid (85 mg) in 42% yield. ^{1}H NMR (CD_2Cl_2, 300 MHz): δ 8.68 (*d*, 1H, *J* = 1.8 Hz), 7.88-7.84 (*m*, 2H), 7.65-7.60 (*m*, 4H), 7.50 (*d*, 1H, *J* = 3Hz), 7.41-7.37 (*m*, 4H), 7.25 (*d*, 1H, *J* = 10.8 Hz), 7.17 (*d*, 1H, *J* = 3.6 Hz), 2.57 (*s*, 3H), 2.55 (*s*, 3H) ^{13}C NMR (CD_2Cl_2; 75 MHz): δ 149.1, 141.5, 140.9, 140.4, 139.4, 138.2, 137.9, 137.5, 137.3, 136.5, 134.6, 130.2, 128.9, 127.1, 126.3, 125.9, 121.0, 118.8, 16.0, 15.8. Anal. Calcd for $C_{24}H_{20}S_2$: C, 77.38; H, 5.41; S, 17.21. Found: C, 77.21; H, 5.52; S, 17.16.

Synthesis of ((6-methylazulene-5,7-diyl)bis(4,1-phenylene))bis(methylsulfane) (5,7 Az).

5,7 Az was synthesized similarly as for **1,3 Az** with compound **4** (0.10 g, 0.33 mmol), compound **2** (0.12 g, 0.73 mmol), $Pd(PPh_3)_4$ (19 mg, 17 μmol). Compound **5,7 Az** was obtained as a dark blue solid (45 mg) in 35% yield. ^{1}H NMR (CD_2Cl_2, 400 MHz): δ 8.29 (*s*, 2H), 7.82 (*s*, 1H), 7.34-7.26 (*m*, 10H), 2.53 (*s*, 6H), 2.09 (*s*, 3H). ^{13}C NMR (CD_2Cl_2; 100 MHz): δ 145.1, 143.6, 138.0,

137.4, 137.3, 137.1, 137.0, 129.6, 126.4, 117.8, 27.7, 15.6. Anal. Calcd for $C_{25}H_{22}S_2$: C, 77.68; H, 5.74; S, 16.59. Found: C, 77.57; H, 5.81; S, 16.43.

^{1}H NMR and ^{13}C NMR spectra

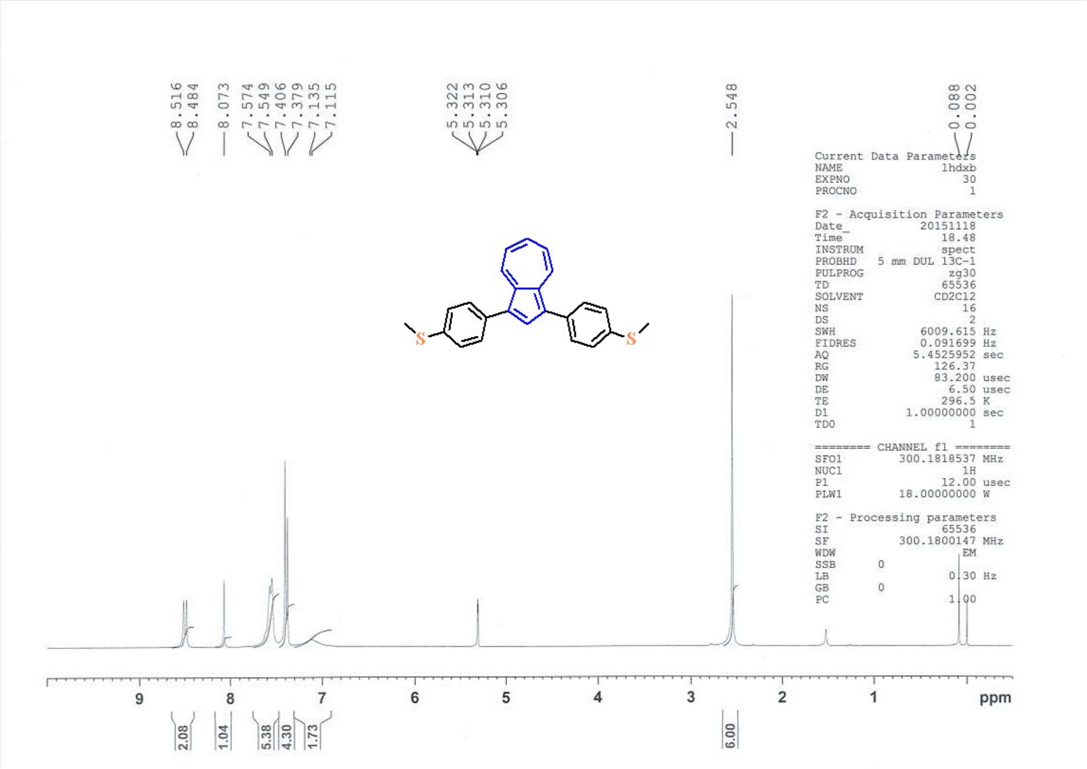

Fig. S1-1 ^{1}H NMR spectra of **1,3 Az**.

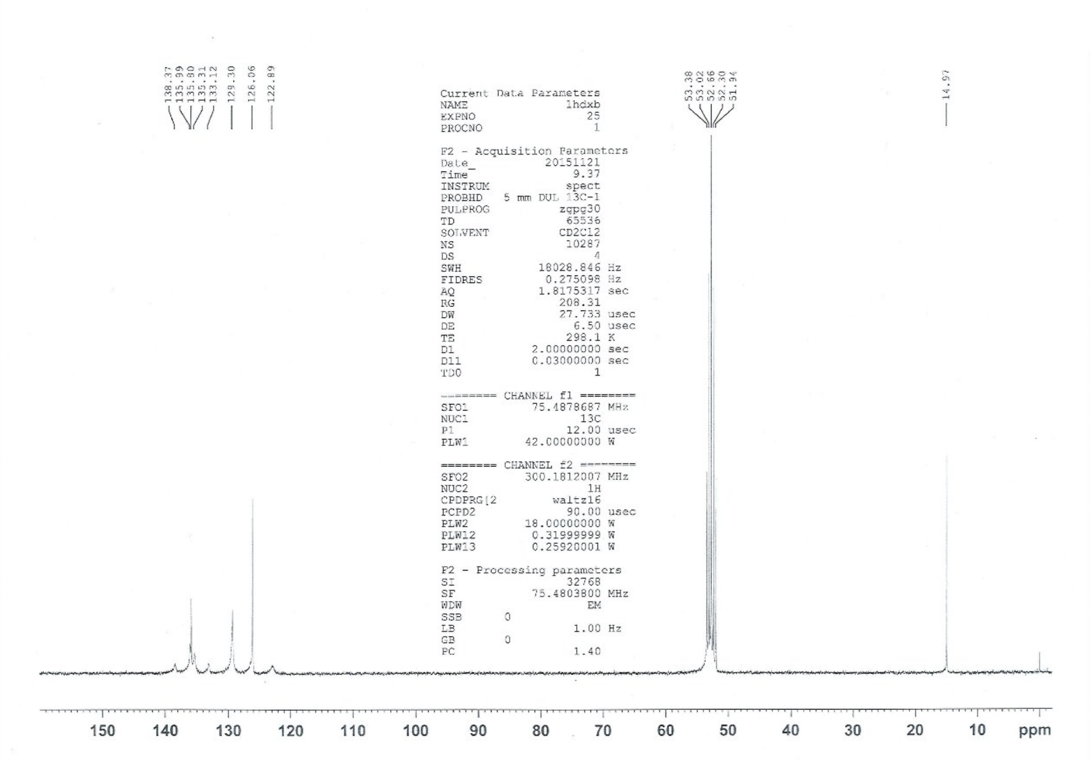

Fig. S1-2 ^{13}C NMR spectra of **1,3 Az**.

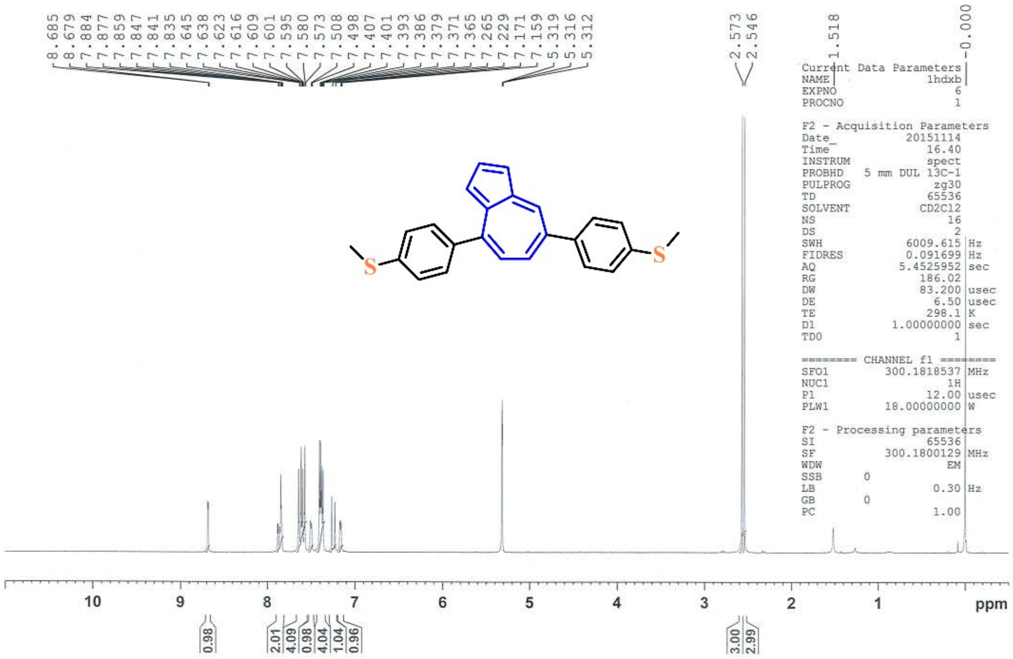

Fig. S1-3 ^{1}H NMR spectra of **4,7 Az**.

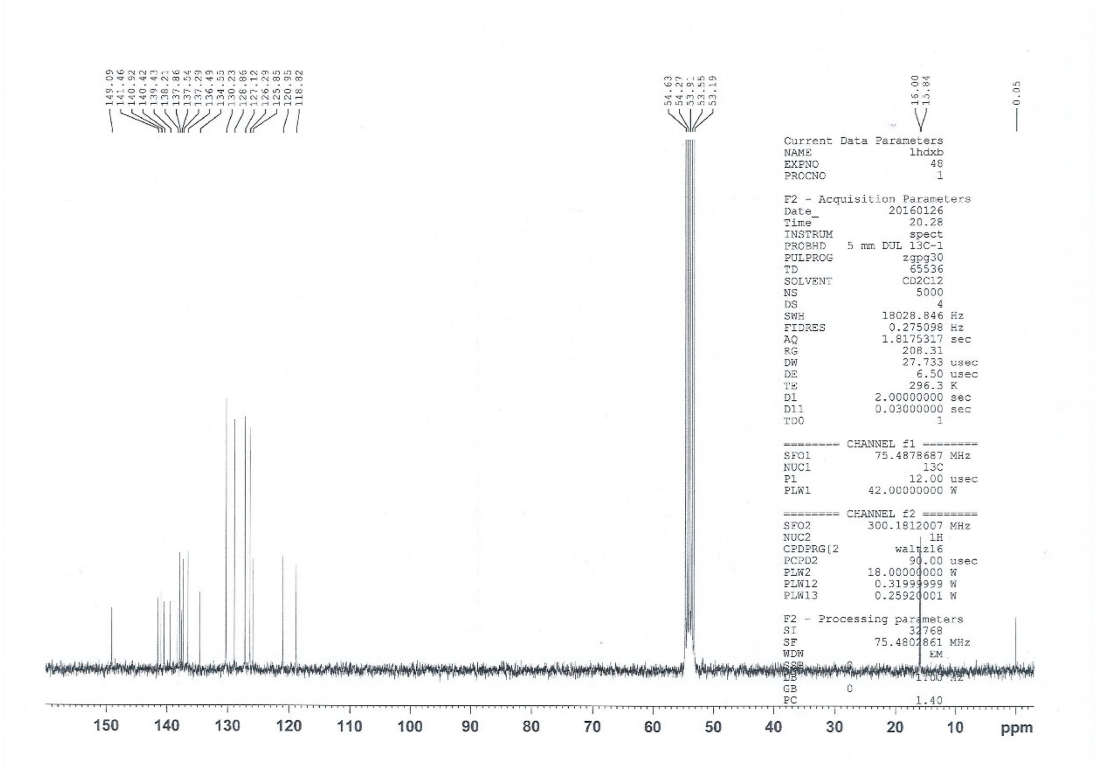

Fig. S1-4 ^{13}C NMR spectra of **4,7 Az**.

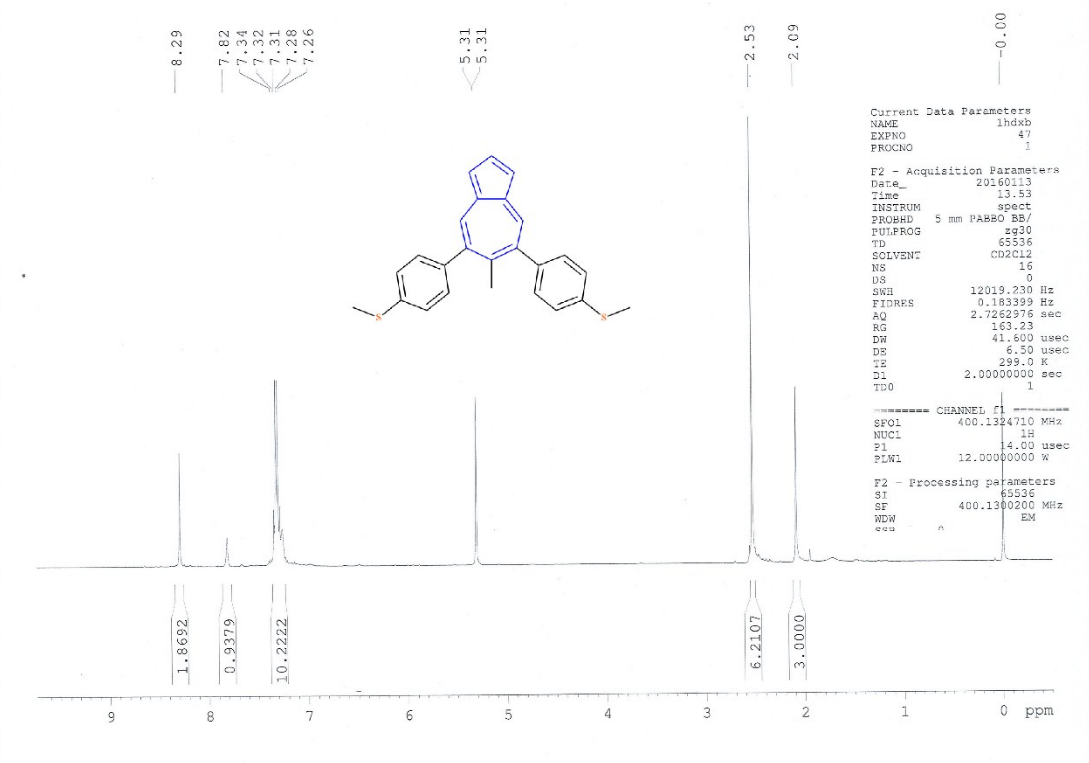

Fig. S1-5 ^{1}H NMR spectra of **5,7 Az**.

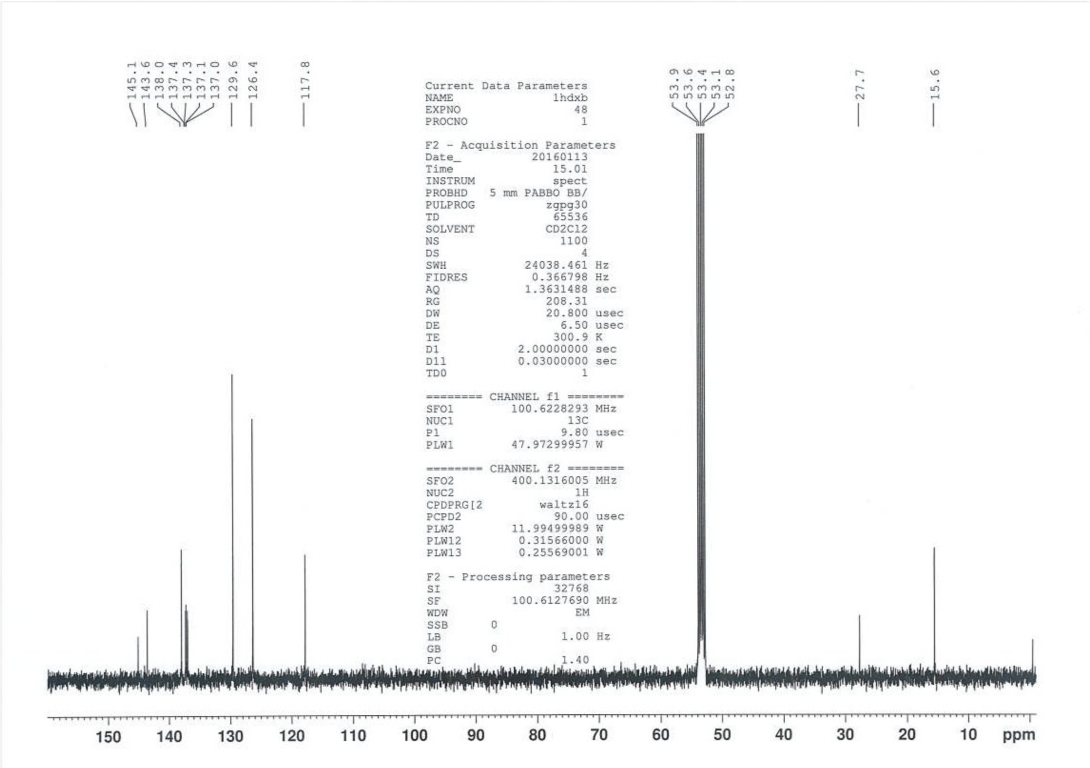

Fig. S1-6 ^{13}C NMR spectra of **5,7 Az**.

2. Single-molecule conductance measurements.

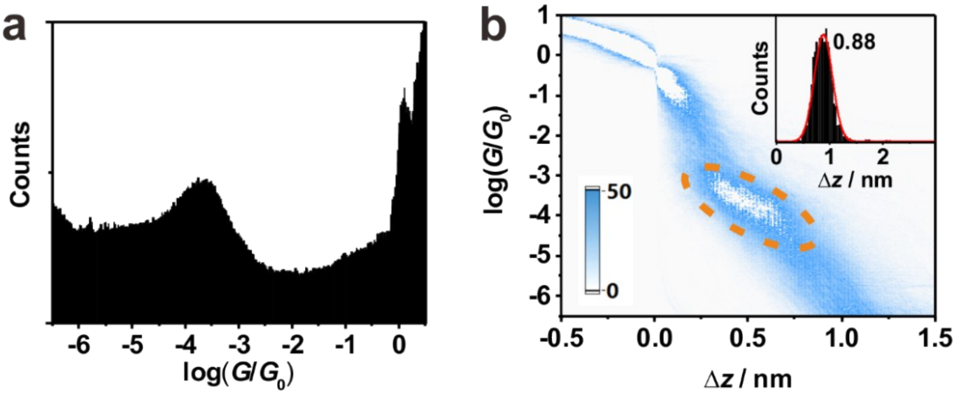

Fig. S2-1 (a) Conductance histograms of **1,3 Az** in 1,2,4-trichlorobenzene and the conductance peak located at G = $10^{-3.6}$ G_0. (b) 2D histogram and stretched distance distribution (inset).

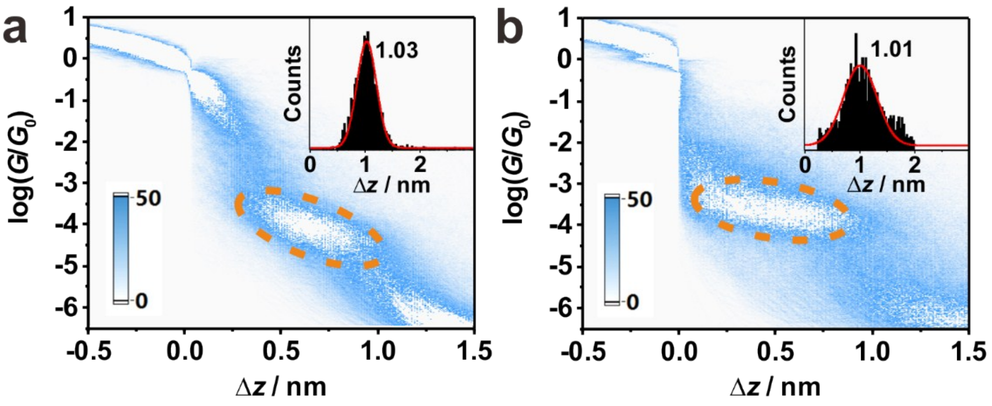

Fig. S2-2 2D histograms and stretched distance distributions (inset) for **4,7 Az** (a) without and (b) withTFA in THF/TMB.

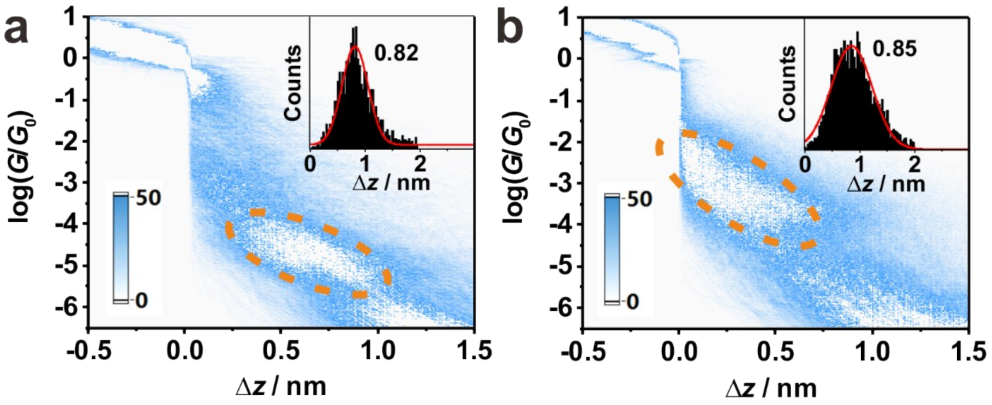

Fig. S2-3 2D histograms and stretched distance distributions (inset) for **5,7 Az** (a) without and (b) with TFA in THF/TMB.

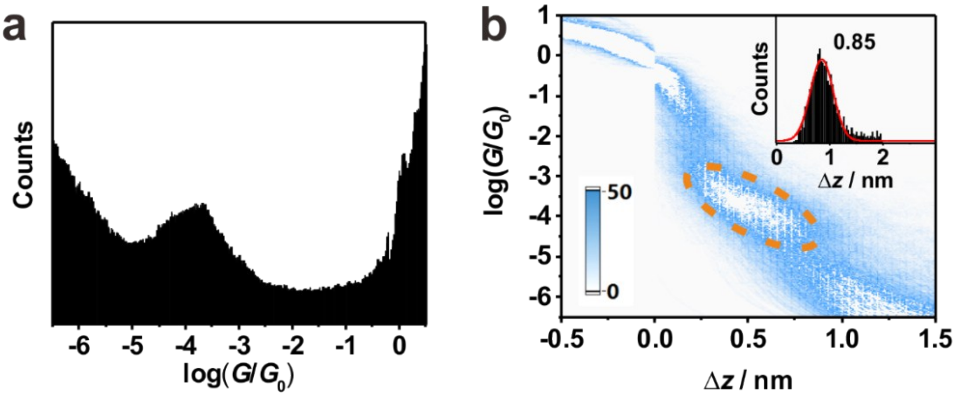

Fig. S2-4 (a) Conductance histograms of **1,3 Az** with 5% (V/V) acetic acid in THF/TMB and the conductance peak located at G = $10^{-3.8}$ G_0. (b) 2D histogram and stretched distance distribution (inset).

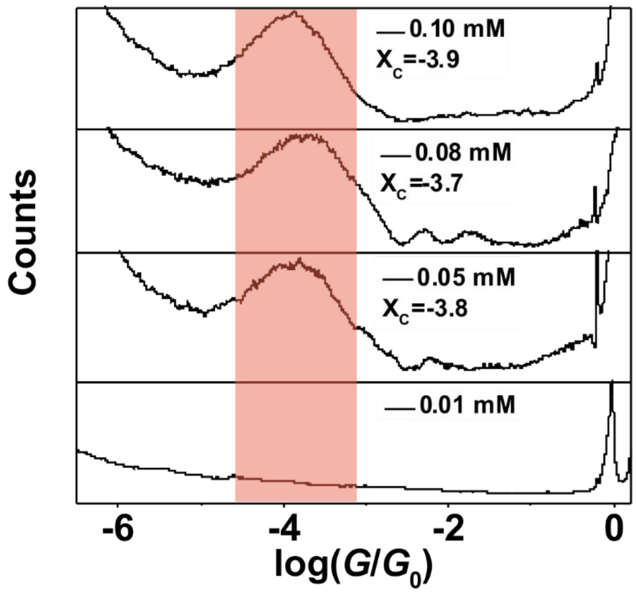

Fig. S2-5 Conductance histograms of **1,3 Az** with different concentrations in THF/TMB.

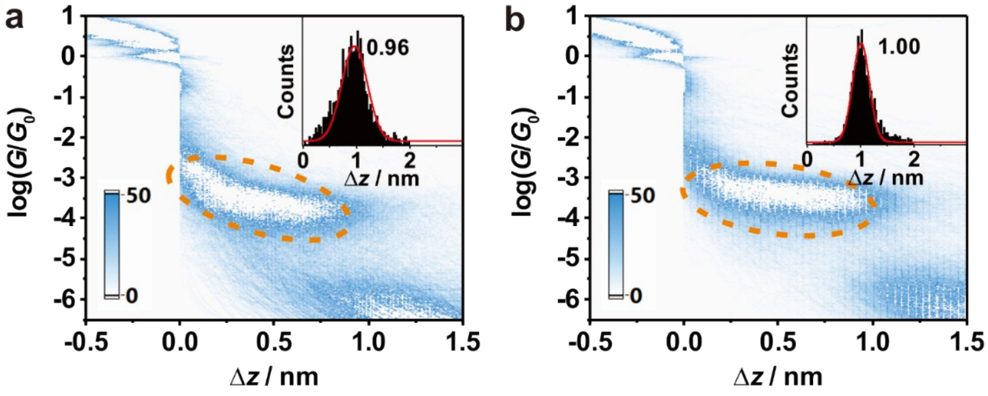

Fig. S2-6 2D histograms and stretched distance distributions (inset) for molecule **Be** (a) without and (b) with TFA in THF/TMB.

3. Calculation

The optimized geometry of each structure shown in Fig. 4a was self-consistently obtained using the SIESTA[3] implementation of density functional theory (DFT). SIESTA employs norm-conserving pseudo-potentials to account for the core electrons and linear combinations of atomic orbitals to construct the valence states. The generalized gradient approximation (GGA) of the exchange and correlation functional is used with the Perdew-Burke-Ernzerhof parameterization (PBE),[4] a double-ζ polarized (DZP) basis set, a real-space grid defined with an equivalent energy cut-off of 250 Ry. The geometry optimization for each structure is performed to the forces smaller than 10 meV/Ang.

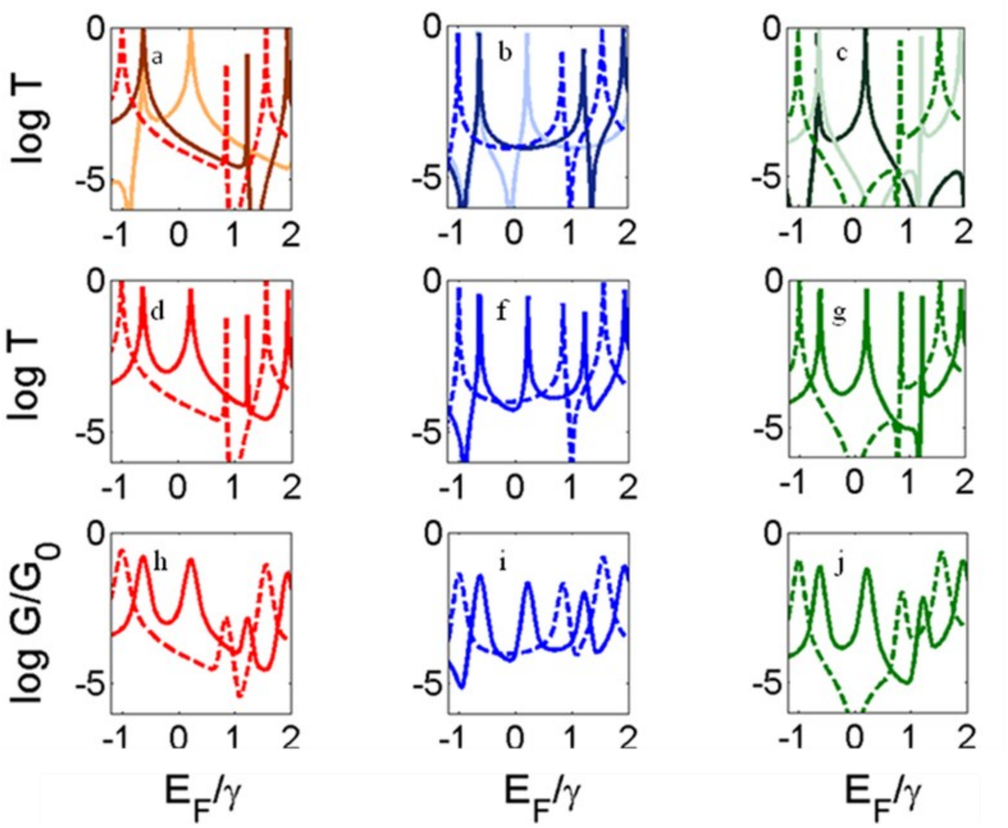

Fig. S3-1 (a-c) TB core transmission coefficients of **1,3 Az, 4,7 Az and 5,7 Az** neutral (dashed line) and protonated (solid line) with spin down and up resonances, (d-g) TB core transmission coefficients of (a-c) neutral (dashed line) and total transmission coefficient ($T_{tot}=T_{up}+T_{down}$) of protonated molecules (solid line). (h-j) room temperature conductance of (d-g).

Figure S3-1 shows results obtained from tight binding modelling of the junctions. These curves were obtained as follows: For the neutral state, for all calculation each site energy was assigned $\varepsilon_0 = 0$ and to obtain the correct gaps the nearest-neighbor couplings for molecule **1,3 Az**, **4,7 Az** and **5,7 Az** are $\gamma = -1.85$, $\gamma = -1.89$, and $\gamma = -2.1$ respectively. These molecular cores in turn

were weakly coupled to single channel leads by matrix elements -0.1. For the tight-binding Hamiltonian parameters of the leads, we chose the site energy ε = 0 and nearest neighbour couplings γ =-1. The transmission coefficient T(E) was computed using the GOLLUM code[5]. For the protonated state, site energies for spin up and down differ by 0.4 eV and the couplings were the same as the neutral molecules.

The above model captures the effect of spin splitting within the junction in the protonated state, even though the isolated non-protonated and protonated molecule is not paramagnetic. This is illustrated by the DFT-predicted spin up and spin down densities of states of the isolated non-protonated molecule and the protonated molecule in the presence of the TFA counterion shown in figure S3-2, which shows that indeed they are identical.

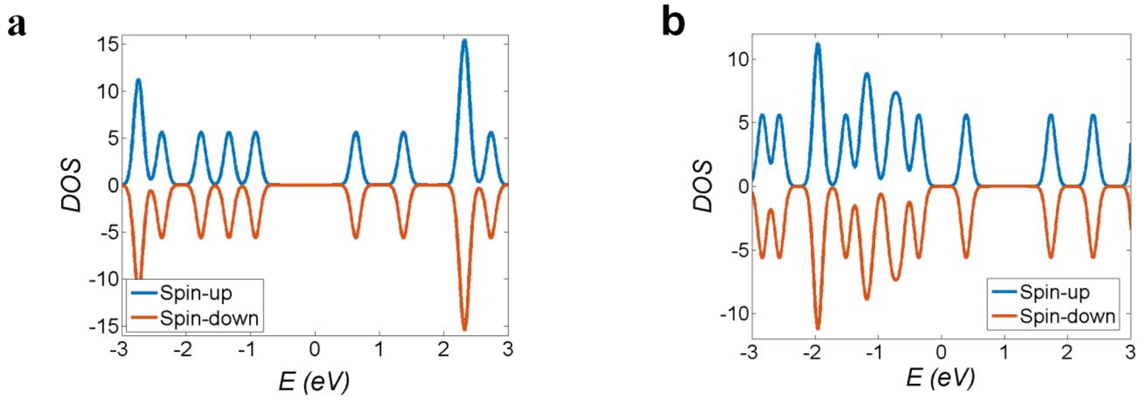

Fig. S3-2 Densities of states of a) isolated non-protonated **1,3 Az** molecule b) isolated protonated **1,3 Az** molecule

Furthermore when placed between gold electrodes, the non-protonated molecule remains non-spin-polarised. This is demonstrated by Figure S3-3c below, which shows plots of the transmission functions of up and down spins, obtained using DFT, for the non-protonated junction. As expected the transmission functions of the non-protonated molecule are independent of spin.

However in the protonated state, when inside a gold junction, charge transfer leads to spin splitting. For the junction in figure S3-3b, consisting of a TFA counterion and the protonated molecule connected to gold electrodes, due to the charge transfer from the molecule to the gold, spin-splitting occurs and the difference between the number (ie Mulliken charge) of spin up and spin down electrons on the azulene is ΔQ≈0.21. Consequently, as shown in figure S3-3d, the transmission functions of the different spins are no longer equal. Figure S3-4 below shows the local spin-dependent densities of states of the protonated junction in two narrow energy windows centred on the HOMO and LUMO transmission resonances. As expected these resonances correspond to two distinct spins.

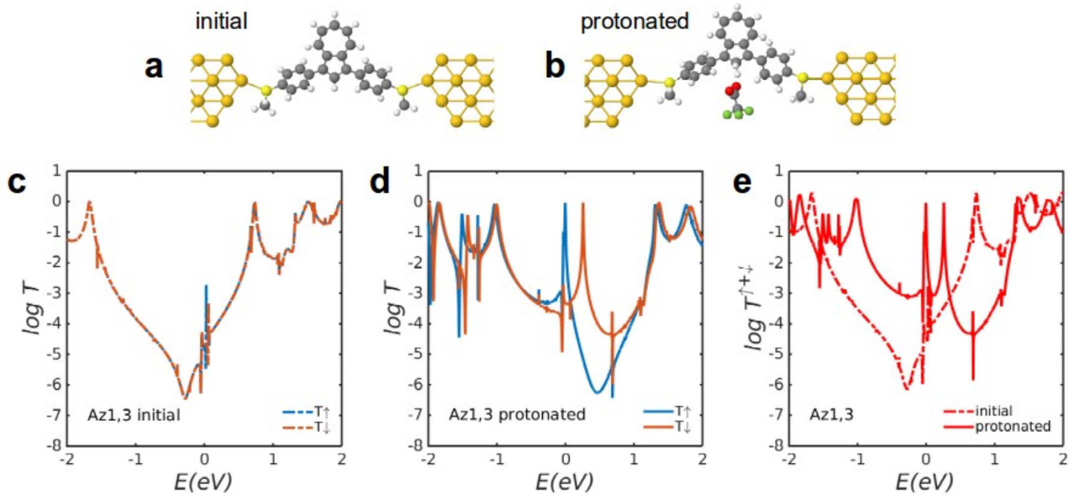

Fig. S3-3 Transmission function T as a function of electron energy *E* obtained from the DFT mean field Hamiltonian. Molecular structure of the junction with **1,3 Az** connectivity in (a) non-protonated and (b) protonated states. The differences between the number (ie Mulliken charge) of spin up and spin down electrons on the **1,3 Az** is $\Delta Q \approx 0$ and $\Delta Q \approx 0.21$ for initial and protonated states. From (c) it is apparent that in the initial non-protonated state the system is not spin polarised. (d) In the protonated state, due to charge transfer from the molecule to the gold, spin-splitting occurs. (e) In agreement with our TB calculation the new HOMO-LUMO gap is smaller than the initial one and arises from the spin-up HOMO and spin down LUMO.

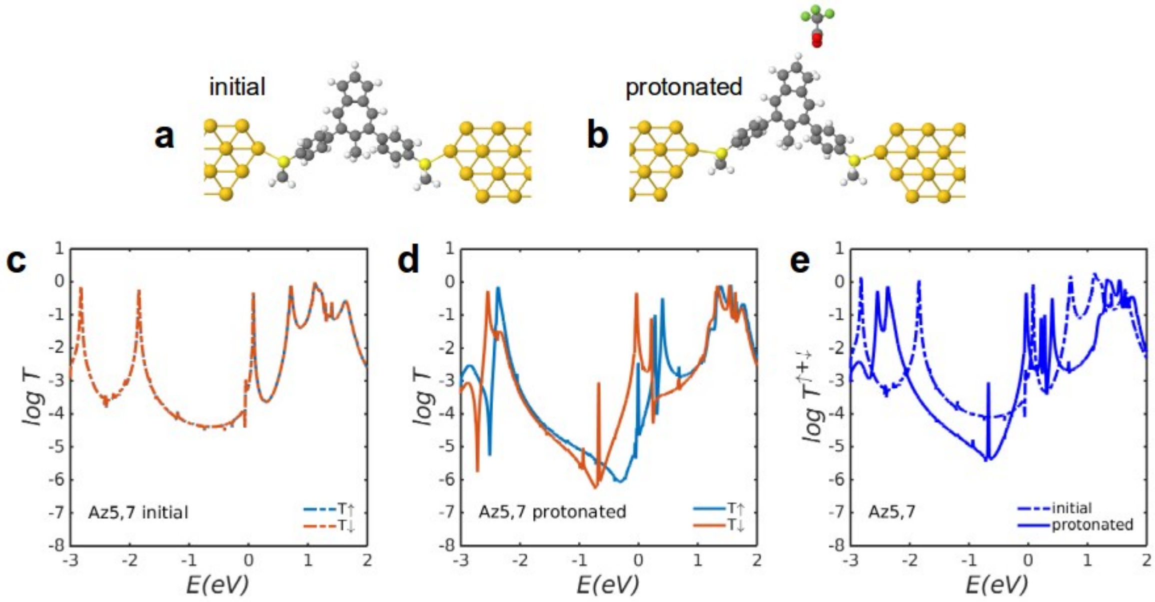

Fig. S3-4 Transmission function T as a function of electron energy *E* obtained from the DFT mean field Hamiltonian. Molecular structure of the junction with 5,7 Az connectivity in (a) non-protonated and (b) protonated states. From (c) it is apparent that in the initial non-protonated state the system is not spin polarised. (d) In the protonated state, due to charge transfer from the molecule to the gold, spin-splitting occurs. (e) In agreement with our TB calculation the new

HOMO-LUMO gap is smaller than the initial one and arises from the spin-up HOMO and spin down LUMO.

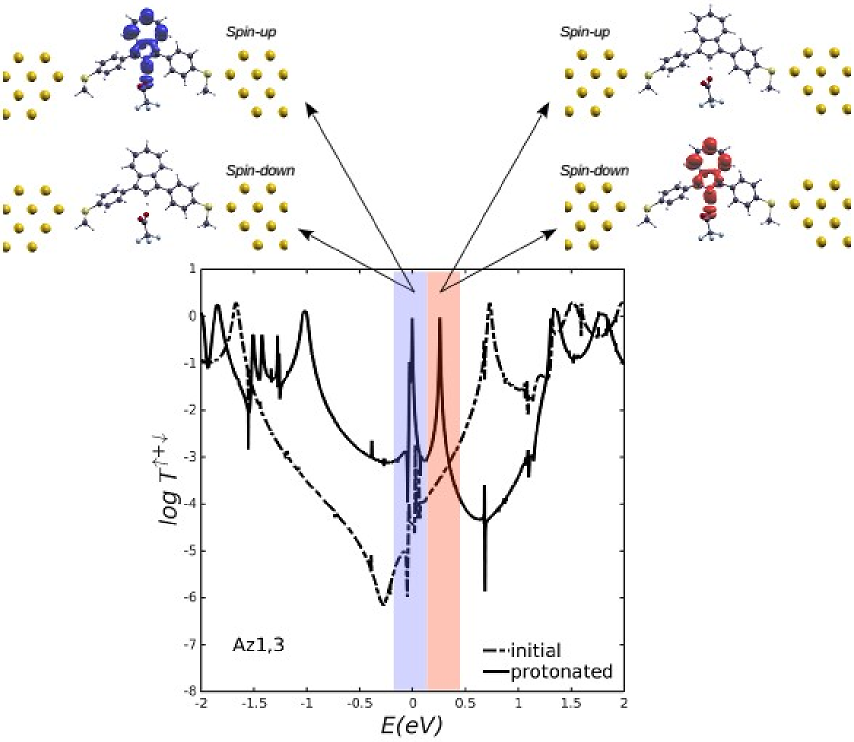

Fig. S3-5 The local spin-dependent densities of states of the **1,3 Az** protonated junction in two narrow energy windows centred on the HOMO and LUMO transmission resonances.

Of course the precise magnitude of the splitting depends on the position of the counterion and in a real junction, an ensemble of positions would be sampled. Our TB model is chosen to capture the key feature of this splitting, namely that the HOMO-LUMO gap of the protonated molecule in a junction is smaller than the initial one.

A limitation of our work is that due to our finite available compute resources, we are only able to model the most stable binding configuration and not a whole ensemble of positions. Another limitation is that there currently exists no theory, which could predict accurately the position of the Fermi energy (E_F) relative to frontier orbitals and therefore we can merely state that with a judicious choice of E_F, experiment is consistent with theory. On the other hand, protonation is generally expected introduce a negative electrostatic potential in the vicinity of the molecule, which causes the HOMO to increase in energy and move closer to the Fermi energy. This effect is independent of the precise location of E_F and for a wide range of Fermi energies, leads to an increase in conductance, as we observe experimentally.

References

1 Y. C. Lu, D. M. Lemal and J. P. Jasinski, J. Am. Chem. Soc., 2000, 122, 2440-2445.

2 J. Xia, B. Capozzi, S. Wei, M. Strange, A. Batra, J. R. Moreno, R. J. Amir, E. Amir, G. C. Solomon, L. Venkataraman and L. M. Campos, Nano Lett., 2014, 14, 2941-2945.

3 J. M. Soler, E. Artacho, J. D. Gale, A. Garcia, J. Junquera, P. Ordejon and D. Sanchez-Portal, J. Phys-Condens. Mat., 2002, 14, 2745-2779.

4 J. P. Perdew, K. Burke and M. Ernzerhof, Phys. Rev. Lett., 1996, 77, 3865-3868.

5 J. Ferrer, C.J. Lambert, V.M. García-Suárez, D. Zs. Manrique, D. Visontai, L. Oroszlany, R. Rodríguez-Ferradás, I.Grace, S.W.D. Bailey, K. Gillemot, H. Sadeghi, L.A. Algharagholy, New J. Phys. 2014, 16, 093029,
